# Translation and cross-cultural adaptation of the *“Protocolo de Avaliação Miofuncional Orofacial MBGR”* from Brazilian Portuguese into English

**DOI:** 10.1371/journal.pone.0295387

**Published:** 2023-12-04

**Authors:** Nayara Ribeiro da Silva, Giédre Berretin-Felix, Carlos Ferreira Santos, Michelle Suzanne Bourgeois

**Affiliations:** 1 Department of Speech-Language Pathology and Audiology, Bauru School of Dentistry, University of São Paulo, Bauru, São Paulo, Brazil; 2 Department of Biological Sciences, Bauru School of Dentistry, University of São Paulo, Bauru, São Paulo, Brazil; 3 Department of Communication Sciences and Disorders, University of South Florida, Tampa, Florida, United States of America; College of Dentistry, Jouf University, SAUDI ARABIA

## Abstract

In health-related research, an increasing number of clinical assessment tools are translated and cross-culturally adapted for cross-national and cross-cultural studies and comparisons. However, when translating and cross-culturally adapting clinical assessment tools for use across new countries, cultures, or languages, we must follow a thorough method to reach semantic, idiomatic, experiential, and conceptual equivalences between translated and original versions. Therefore, in this study, we translated and cross-culturally adapted the *Protocolo MBGR* (Marchesan, Berretin-Felix, Genaro, and Rehder) from Brazilian Portuguese into English, following international guidelines, and named it “MBGR Protocol.” To verify its content validity, we used the Content Validity Index. Results indicated excellent content validity: a Scale-Content Validity Index of 0.96 and 97% of all translation units with an Item-Content Validity Index of 1.00. Also, to prove its face validity and confirm whether it worked in the target population’s linguistic-cultural setting, we used it with 35 subjects. Again, results demonstrated excellent face validity: in the pretest, 91% of all translation units were considered comprehensible and clear; in the pilot test, 98% of all translation units were considered comprehensible and clear. Thus, we concluded that the MBGR Protocol is promising to enhance the uptake of studies in Orofacial Myology worldwide and support researchers and health professionals in assessing and diagnosing orofacial myofunctional disorders in children, adolescents, adults, and the elderly. Also, it may support evidence-based practice and assist in standardizing assessment and diagnostic criteria. The MBGR Protocol should have its psychometric properties tested before being used in clinical practice or scientific research. Therefore, future studies are needed, and collaborations among researchers from South and North American countries are encouraged to create an international network and advance with knowledge and skills in the Orofacial Myology discipline.

## Introduction

As a field of study and research, Orofacial Myology aims to promote an understanding of the stomatognathic system [1]. The stomatognathic system consists of a complex and interdependent set of organs, tissues, and structures and performs functions such as phonation, chewing, breathing, and swallowing [1, 2]. Orofacial Myology, therefore, is the specialty area dedicated to the study, prevention, assessment, diagnosis, and treatment of orofacial myofunctional disorders (OMDs), that is, of congenital or acquired changes in organs, tissues, and structures of the mouth, face, and neck regions, and stomatognathic functions [1].

Common signs and symptoms of OMDs may include (a) open mouth resting posture; (b) structural abnormalities, such as restricted lingual frenulum; (c) dental abnormalities, such as anterior or posterior open bite, excessive anterior overjet, and underbite; (d) abnormal tongue resting posture, such as tongue forward; (e) abnormal breathing patterns; (f) distorted productions of /s/ and /z/ sounds, often with an interdental lisp; (g) abnormal linguodental articulatory placement for /t/, /d/, /l/, and /n/ sounds; (h) drooling and poor oral control; (i) nonnutritive sucking habits, such as pacifier, finger, thumb, or tongue sucking; (j) lack of a consistent linguopalatal seal during liquid, solid, and saliva swallows; (k) chewing difficulties; and (l) asymmetry of the face [3–16]. Therefore, OMDs can negatively impact orofacial postures and functions, leading to additional health problems and significantly lowering patients’ quality of life [6, 17].

In Brazil, Orofacial Myology consolidated itself as a field of study and research through the interdisciplinarity between Speech-Language Pathology and Dentistry, especially Orthodontics [18]. In 1976, Brazilian speech-language pathologists (SLPs) developed the earliest national research paper on Orofacial Myology; soon after, in the 90s, research on the subject escalated [18]. In 1996, the Brazilian Federal Council of Speech-Language Pathology and Audiology recognized Orofacial Myology as a specialty of Speech-Language Pathology [18]. So, university graduate and training courses in Orofacial Myology multiplied across the country, and simultaneously, the number of national research papers on the subject also increased [18].

In the United States (US), Orthodontics has also strongly influenced the Orofacial Myology area [19]. In the early 20th century, dentists developed the first national research papers on Orofacial Myology, and in the 60s, SLPs also developed their earliest reports on the subject [19, 20]. However, Orofacial Myology is not recognized as a specialty of Dentistry or Speech-Language Pathology in the US; hence, no North American university graduate or training course offers a degree in the discipline [17, 19, 21]. Hanson and Mason explain that professionals from different health areas, such as dentists, SLPs, physical therapists, and doctors, can work as orofacial myologists if certified by the Academy of Orofacial Myofunctional Therapy (AOMT) or the International Association of Orofacial Myology (IAOM) [17, 22]. Dentists, for example, can look at patients’ teeth and jaw movements; SLPs can assess patients’ speech and how they swallow and breathe; physical therapists can focus on improving the strength, positioning, and coordination of patients’ mouth and neck muscles; and doctors can test for allergies and check patients’ tonsils and adenoids. Thus, due to the decentralization of the area, interest in developing scientific research on Orofacial Myology in the United States has only emerged in recent years [19].

Research topics in Orofacial Myology are various. For example, in Brazil, since 2002, the Orofacial Myology Committee of the Brazilian Society of Speech-Language Pathology and Audiology has been committed to conducting scientific research on the development of orofacial myofunctional assessment tools aiming at standardizing clinical assessment in Orofacial Myology [23]. Likewise, in the United States, since 2005, the American Speech-Language-Hearing Association (ASHA) has commended that health professionals use clinical assessment tools to ensure evidence-based practice and support standardizing assessment and diagnostic criteria in Orofacial Myology [24]. Felício and colleagues explain that a standardized clinical assessment in Orofacial Myology is imperative as it identifies subjects’ OMDs and determines the classification and frequency of these disorders in different pathologies, age groups, and populations [2].

Accordingly, some Brazilian researchers have developed clinical assessment tools that permit relating orofacial myofunctional conditions to numerical scores or scales [6, 7, 25–30]. So, they have developed four orofacial myofunctional assessment tools for individuals over six years old: the Protocol of Orofacial Myofunctional Evaluation with Scores (OMES) [6] and its expanded version [7]; and the *Protocolo de Avaliação Miofuncional Orofacial MBGR* [25, 30] and its updated version [26]. Also, they have developed two orofacial myofunctional assessment tools for infants and preschoolers: the Expanded Protocol of Orofacial Myofunctional Evaluation with Scores for Nursing Infants (OMES-E Infants) [27] and the MMBGR Protocol—Infants and Preschoolers [28, 29].

In the United States, still due to the decentralization of clinical practice and scientific research on Orofacial Myology, clinical assessment tools for assessing and diagnosing OMDs come from different health areas [31, 32]. So, as an immediate result, North American orofacial myologists can utilize clinical assessment tools common to dentists, dental hygienists, SLPs, doctors, and other disciplines touching the stomatognathic system [32]. As a secondary result, however, they have no “gold standard” clinical assessment tools for assessing and diagnosing OMDs [32].

Considering the above-mentioned, researchers acknowledge Brazil as a leader in developing scientific research and clinical assessment tools in Orofacial Myology and acquiesce that Brazilian technical and scientific knowledge on the subject is of worldwide interest [19, 21]. Even today, Brazil is the first and only country to have the Federal Council recognizing Orofacial Myology as a specialty area of Speech-Language Pathology [21]. Thus, writing and publishing Brazilian technical and scientific knowledge in Orofacial Myology only in Portuguese is not conducive as it restricts it from the international scientific scenario [33]. Also, since the second half of the 20th century, English has been the *lingua franca* of science [33].

Statistical data validate the last statement. By analyzing the journals indexed in the Web of Science database in 2020, it is possible to observe that only 7.7% of German papers were written in German, and only 5.3% of French papers were written in French [33]. Also, only 2.3% of Italian papers were written in Italian, and only 3% of Portuguese papers were written in European Portuguese [33]. In Spanish-speaking countries, these percentages are slightly higher: 12% of Mexican papers, 13% of Spanish papers, 16% of Chilean papers, and 20% of Argentinian, Colombian, and Peruvian papers were written in Spanish [33]. The data in Brazil are closer to the rest of Latin America: 12% of Brazilian research papers were written in Brazilian Portuguese [33]. When this analysis is extended, the result is forthright: 90% of the science published on the Web of Science in the last twenty years is written in English [33].

Accordingly, some Brazilian researchers have also translated their scientific research and clinical assessment tools in Orofacial Myology into English [6, 7, 10, 27–29, 34–38]. Therefore, this study aimed to translate and cross-culturally adapt the *Protocolo de Avaliação Miofuncional Orofacial MBGR* [26] from Brazilian Portuguese into English, following the guidelines proposed by Beaton and colleagues [39]. As a result, this study may enhance the uptake of studies in Orofacial Myology worldwide and support researchers and health professionals in assessing and diagnosing OMDs in children, adolescents, adults, and the elderly. Also, it may support evidence-based practice and assist in standardizing assessment and diagnostic criteria in Orofacial Myology.

## Materials and methods

This descriptive and methodological study addresses the translation and cross-cultural adaptation of the *Protocolo MBGR* from Brazilian Portuguese into English.

### Ethical considerations

This study was approved by the Institutional Review Board of the Bauru School of Dentistry—University of São Paulo (USP). Also, written permission to translate and cross-culturally adapt the *Protocolo MBGR* into English was obtained from its developers, and written informed consent was obtained from all participants.

### Protocolo MBGR

The *Protocolo de Avaliação Miofuncional Orofacial MBGR* (Marchesan, Berretin-Felix, Genaro, and Rehder) assesses and diagnoses OMDs in children, adolescents, adults, and the elderly; it includes an Orofacial Myofunctional Clinical History and an Orofacial Myofunctional Clinical Examination with Scores [26]. The Orofacial Myofunctional Clinical History collects information on the patient’s personal and medical history, and the Orofacial Myofunctional Clinical Examination with Scores examines the patient’s mouth, face, and neck regions and stomatognathic functions [26].

The assessment tool allows for determining the normal reference values of OMDs in healthy individuals, analyzing the characteristics of OMDs in patients, and differentiating healthy individuals from patients. Additionally, its scoring system permits relating orofacial myofunctional conditions to numerical scores and determining the severity of OMDs [26].

The *Protocolo MBGR* has been widely used in Brazilian SLPs’ clinical practice and national scientific research on Orofacial Myology [9, 35, 36, 40–56]. Moreover, it was validated for individuals with temporomandibular disorders [38] and adapted and validated for individuals with cleft lip and palate [37].

### Process of translation and cross-cultural adaptation of the *Protocolo MBGR* into English

The process of translation and cross-cultural adaptation of the *Protocolo MBGR* into English followed the guidelines recommended by Beaton et al. [39] and consisted of six stages as follows:

### Translation

Two native English translators independently translated the original Portuguese version of the *Protocolo MBGR* into English (T1 and T2). The T1 translator had extensive experience translating health-related content, including content in speech-language pathology, and was aware of the purpose of the study. Conversely, the T2 translator had little experience translating health-related content and was unaware of the purpose of the study.

### Synthesis of the translations

The translators and a recording observer synthesized the translations (T1 and T2) into one common translation (T12). The recording observer was a native Portuguese translator with extensive experience translating health-related content, including content in speech-language pathology.

First, they compared the translations (T1 and T2) based on the original Portuguese version of the *Protocolo MBGR* and highlighted the translation units considered dubious or debatable. Next, the translators, the recording observer, and the researcher met to discuss, review, and adjust all highlighted translation units. The researcher was also a native Portuguese translator with extensive experience translating health-related content, including content in speech-language pathology.

### Back-translation

Working from T12 and blinded to the original Portuguese version of the *Protocolo MBGR*, two native Portuguese translators independently produced two back-translations (BT1 and BT2) into Brazilian Portuguese. Both back-translators had extensive experience translating health-related content, including content in speech-language pathology, and were aware of the purpose of the study.

### Expert committee

The four translators, the recording observer, and two SLPs analyzed all translations (T1, T2, T12, BT1, and BT2), established the pre-final English version of the assessment tool for field testing, and proved its content validity. The SLPs were native Portuguese speakers fluent in English.

First, the experts individually analyzed all translations considering semantic, idiomatic, experiential, and conceptual equivalences [39] and scored each translation unit as “1 = not equivalent,” “2 = somewhat equivalent,” “3 = quite equivalent,” or “4 = highly equivalent,” according to the Content Validity Index (CVI) [57]. They also suggested better translations for all translation units scored as “1” or “2.”

The CVI allowed us to evaluate each translation unit independently by calculating its Item-Content Validity Index (I-CVI). To calculate the I-CVI, we divided the cumulative frequency of scores “3” and “4” of each translation unit by the total number of experts. It also allowed us to determine an overall average of the I-CVI scores for the assessment tool by calculating its Scale-Content Validity Index (S-CVI). To calculate the S-CVI, we added all translation units scored as “3” and “4” by all experts and divided this sum by the total number of translation units. As recommended, an I-CVI value of 0.78 or above and an S-CVI value of 0.90 or above were considered acceptable [57, 58].

Next, the experts and the researcher met to discuss, review, and adjust all translation units scored as “1” or “2” and all translation units with an I-CVI lower than 0.78. A methodologist, a native Portuguese translator with extensive experience translating health-related content, including content in speech-language pathology, mediated this meeting.

### Test of the pre-final version

We used the pre-final English version of the *Protocolo MBGR* with subjects of the study’s target population, established its final English version, and proved its face validity. This stage was held at the University of South Florida (USF) in Tampa, United States of America, and was divided into two phases: pretest and pilot test.

In the pretest, we tested the pre-final English version of the *Protocolo MBGR*. To do so, 20 SLPs analyzed the pre-final English version of the assessment tool to verify whether it worked in their linguistic-cultural setting. We asked them to provide feedback on its clarity and interpretability. So, they read the pre-final English version of the assessment tool and searched for misunderstandings. Then, they highlighted the translation units that did not make sense to them and the ones that made sense to them but sounded weird or unusual and for which they could think of a better term. They also wrote comments explaining why they highlighted each translation unit. The SLPs were native English speakers. Fifteen of them were enrolled in some USF Graduate Programs, and five of them were USF professors.

Next, two speech-language pathology professors and the researcher met to discuss, review, and adjust all highlighted translation units. One professor was a native English speaker. The other professor was a native Portuguese speaker fluent in English who had been living and teaching in the United States of America for about ten years.

In the pilot test, we retested the pre-final English version of the *Protocolo MBGR*. To do so, 15 other SLPs analyzed the adjusted pre-final English version of the assessment tool to verify whether it worked in their linguistic-cultural setting. We asked them to provide feedback on its clarity and interpretability. So, they participated in a training workshop conducted by one of the developers of the *Protocolo MBGR* and approved by ASHA as an ASHA Continuing Education program. They worked in pairs and administered the adjusted pre-final English version of the *Protocolo MBGR* to each other.

Meanwhile, they searched for misunderstandings. They highlighted the translation units that did not make sense to them and the ones that made sense to them but sounded weird or unusual and for which they could think of a better term. They also wrote comments explaining why they highlighted each translation unit. The SLPs were native English speakers and were all ASHA members.

Then, the two speech-language pathology professors and the researcher met to discuss, review, and adjust all highlighted translation units.

### Submission of documentation to the coordinating committee for appraisal of the adaptation process

A coordinating committee of two USP professors proofread the final English version of the assessment tool and appraised the reports on all the methodological stages of its translation and cross-cultural adaptation process. One professor was a native Portuguese speaker fluent in English. The other professor was a native Portuguese translator with extensive experience translating health-related content, including content in speech-language pathology.

## Results

The *Protocolo MBGR* was translated and cross-culturally adapted from Brazilian Portuguese into English, according to the guidelines recommended by Beaton et al. [39], and named “MBGR Protocol.”

The assessment tool was developed for the exclusive use of healthcare professionals, such as SLPs, dentists, dental hygienists, and doctors. Therefore, considering the level of education and information of its administrators, its language is quite specialized. That said, during its translation and cross-cultural adaptation procedure, we strived to ensure that the language of its final English version was also specialized.

### Process of translation and cross-cultural adaptation of the *Protocolo MBGR* into English

#### Translation

We observed that T1 and T2 achieved similar results but differed significantly regarding specialized terminology.

The two native English translators independently translated 1,618 translation units from Brazilian Portuguese into English. Of these 1,618 translation units, 1,148 (71%) were translated the same in T1 and T2, and 470 (29%) were translated differently. Most translation units translated differently were different English equivalents for one same Portuguese translation unit; for example, “dairy” and “milk and derivatives” were English equivalents for *“leite e derivados*,*”* and “breathing” and “respiration” were English equivalents for *“respiração*.*”* Additionally, “chewing” and “mastication” were English equivalents for *“mastigação*,*”* and “observe the patient standing barefoot” and “observe the patient standing without shoes” were English equivalents for *“observar o paciente em pé e sem calçado*.*”*

However, some of these translation units differed significantly regarding specialized terminology. For example, the T1 translator translated *“postura anteriorizada da cabeça”* as “forward head posture” (specialized terminology), and the T2 translator translated it as “anterior head posture” (literal translation). Similarly, the T1 translator translated *“postura anteriorizada dos ombros”* as “rounded shoulders” (specialized terminology), and the T2 translator translated it as “anterior shoulder posture” (literal translation).

Thus, we assumed that, when it came to specialized terminology, the experience and awareness of the translator were indispensable. Also, we presumed that the translations produced by the experienced and informed translator provided appropriate and reliable cross-cultural adaptations from a clinical perspective.

#### Synthesis of the translations

Because T1 and T2 achieved similar results, the translators and the recording observer synthesized them into T12 with no significant issues; few adjustments were necessary.

Of those 1,148 translation units translated the same in T1 and T2, 18 (1%) needed adjustments. Also, of those 470 translation units translated differently, 64 (4%) needed adjustments. Thus, the translators, the recording observer, and the researcher adjusted some translation units to eliminate misunderstandings; for example, both translators translated *“amamentação”* as “breastfeeding,” but it referred to both breastfeeding and bottle-feeding, so they changed it to “breast and bottle feeding.”

They also adjusted some translation units to ensure the use of specialized terminology. For example, the T1 translator translated *“zumbido”* as “ear ringing,” and the T2 translator translated it as “buzzing.” Still, they changed it to “tinnitus” because, although “ear ringing” and “buzzing” were English equivalents for *“zumbido*,*”* “tinnitus” was the recurrent specialized terminology in speech-language pathology and audiology contents.

Furthermore, of those 470 translation units translated differently, 335 (21%) remained as in T1, and 60 (4%) as in T2. Most of these translation units remained as in T1 because, again, some of them differed significantly regarding specialized terminology; for example, the T1 translator translated *“má oclusão”* as “malocclusion” (specialized terminology), and the T2 translator translated it as “bad occlusion” (literal translation). Similarly, the T1 translator translated *“asas do nariz”* as “nasal alae” (specialized terminology), and the T2 translator translated it as “nose wings” (literal translation).

Therefore, considering the level of education and information of the administrators of the assessment tool, we considered it necessary to ensure the use of specialized terminology in T12. Furthermore, we concluded that the translation produced by the experienced and informed translator provided appropriate and reliable cross-cultural adaptations from a clinical perspective.

#### Back-translation

We observed that BT1 and BT2 achieved similar results, yet by comparing them with the original Portuguese version of the *Protocolo MBGR*, we found misunderstandings in T12.

Working from T12 and blinded to the original Portuguese version of the assessment tool, the two native Portuguese translators independently translated 1,618 translation units from English into Brazilian Portuguese. Of these 1,618 translation units, 1,174 (72.5%) were translated the same in BT1 and BT2, and 444 (27.5%) were translated differently. Most translation units translated differently were different Portuguese equivalents for one same English translation unit; for example, *“frênulo lingual”* and *“frênulo da língua”* were Portuguese equivalents for “tongue frenulum,” and *“hábitos posturais”* and *“hábitos de postura”* were Portuguese equivalents for “posture habits.” Also, *“some todas as pontuações”* and *“some todos os pontos”* were Portuguese equivalents for “add all scores,” and *“comissura dos lábios”* and *“comissura labial”* were Portuguese equivalents for “commissure of the lips.”

However, by comparing BT1 and BT2 with the original Portuguese version of the *Protocolo MBGR*, we found misunderstandings in T12. First, the T1 translator translated *“espirros em salva”* as “uncontrolled sneezing,” and it remained as “uncontrolled sneezing” in T12. Then, the BT2 translator translated “uncontrolled sneezing” as *“espirros sem controle*.*” “Espirros sem controle”* in BT2 was equivalent to “uncontrolled sneezing” in T12, but *“espirros sem controle”* in BT2 was not equivalent to *“espirros em salva”* in the original Portuguese version of the *Protocolo MBGR*. Therefore, we observed that the BT2 translator translated *“espirros sem controle”* correctly, but “uncontrolled sneezing” in T12 needed to be adjusted because it was not equivalent to *“espirros em salva”* in the original Portuguese version of the assessment tool. So, we changed it to “repeated sneezing.”

Similarly, first, the T1 translator translated *“reprovações (escolares)”* as “failures,” and it remained as “failures” in T12. Then, the BT1 translator translated “failures” as *“falhas*.*” “Falhas”* in BT1 was equivalent to “failures” in T12, but *“falhas”* in BT1 was not equivalent to *“reprovações”* in the original Portuguese version of the *Protocolo MBGR*. Therefore, again, we observed that the BT1 translator translated *“falhas”* correctly, but “failures” in T12 needed to be adjusted because it was not equivalent to *“reprovações”* in the original Portuguese version of the assessment tool. So, we changed it to “grade retentions.”

Therefore, by indicating unclear wording, the back-translation stage was a validity-checking process to ensure that T12 was equivalent to the original Portuguese version of the *Protocolo MBGR*.

### Expert committee

The expert committee established the pre-final English version of the *Protocolo MBGR* for field testing, proved its content validity, and ensured that it attained semantic, idiomatic, experiential, and conceptual equivalences with its original Portuguese version.

The four translators, the recording observer, and the two SLPs individually scored each of the 1,618 translation units as “1 = not equivalent,” “2 = somewhat equivalent,” “3 = quite equivalent,” or “4 = highly equivalent,” according to the CVI. Of these 1,618 translation units, 1,566 (97%) were scored as “3” or “4” by all experts and achieved an I-CVI of 1.00. Also, 39 (2%) translation units were scored as “1” or “2” by at least one expert and achieved an I-CVI of 0.85. Moreover, 13 (1%) translation units were scored as “1” or “2” by multiple experts and achieved an I-CVI lower than 0.78.

As recommended by Polit and Beck [57], the experts and the researcher adjusted all translation units scored as “1” or “2” and all translation units with an I-CVI lower than 0.78. Most adjustments aimed to convert the units of measurement from the metric system to the imperial system; they changed meters and centimeters to feet and inches and kilograms and grams to pounds. Also, they adjusted some translation units to ensure the use of specialized terminology. For example, they changed “reduced nasal use” (literal translation of *“uso reduzido nasal”*) to “hyponasality” (specialized terminology) and “excessive nasal use” (literal translation of *“uso excessivo nasal”*) to “hypernasality” (specialized terminology).

Therefore, we established the pre-final English version of the *Protocolo MBGR* for field testing and confirmed its content validity with an S-CVI of 0.96 and 97% of the translation units with an I-CVI of 1.00. Furthermore, considering the level of education and information of the administrators of the assessment tool, we considered it necessary to ensure the use of specialized terminology in its pre-final English version.

### Test of the pre-final version

All 35 SLPs participating in this stage reported that the pre-final English version of the *Protocolo MBGR* and its adjusted version were comprehensive and clear.

In the pretest, the 20 SLPs provided feedback on the clarity and interpretability of the pre-final English version of the assessment tool. First, they read its 1,618 translation units and searched for misunderstandings. Then, they highlighted the translation units that did not make sense to them and the ones that made sense to them but sounded weird or unusual and for which they could think of a better term. Also, they wrote comments explaining why they highlighted each translation unit. As a result, 145 (9%) translation units were highlighted.

As mentioned, the two speech-language pathology professors and the researcher adjusted all highlighted translation units. Most adjustments aimed to ensure the use of specialized terminology. Considering the translation units related to loudness, for example, they changed “strong” (literal translation of *“forte”*) to “loud” (specialized terminology) and “weak” (literal translation of *“fraco”*) to “quiet” (specialized terminology).

Similarly, they changed “absence of sound production as a baby” (literal translation of *“ausência de produção de sons quando bebê”)* to “delayed babble” (specialized terminology), “took long to start speaking” (literal translation of *“demorou a falar”*) to “delayed onset of speech” (specialized terminology), and “took long to start elaborating sentences” (literal translation of *“demorou a elaborar frases”*) to “delayed onset of language” (specialized terminology).

Then, in the pilot test, the other 15 SLPs provided feedback on the clarity and interpretability of the adjusted pre-final English version of the *Protocolo MBGR*. They participated in a training workshop, and working in pairs, they administered it to each other. Meanwhile, they analyzed its 1,618 translation units and searched for misunderstandings. They highlighted the translation units that did not make sense to them and the ones that made sense to them but sounded weird or unusual and for which they could think of a better term. Also, they wrote comments explaining why they highlighted each translation unit. As a result, 36 (2%) translation units were highlighted.

Again, the two speech-language pathology professors and the researcher adjusted all highlighted translation units. Most adjustments aimed to ensure the use of specialized terminology. Considering the translation units related to voice, for example, they changed “weakness” (literal translation of *“fraqueza”*) to “hypophonia” (specialized terminology) and “muteness” (literal translation of *“mudez”*) to “aphonia” (specialized terminology). Similarly, they adjusted “lateral dominance” (literal translation of *“dominância lateral”*) to “handedness” (specialized terminology).

Therefore, we tested and retested the pre-final English version of the *Protocolo MBGR*, established its final English version, and confirmed its face validity. Furthermore, considering the level of education and information of the administrators of the assessment tool, we considered it necessary to ensure the use of specialized terminology in its final English version.

### Submission of documentation to the coordinating committee for appraisal of the adaptation process

The coordinating committee proofread the final English version of the *Protocolo MBGR* and appraised the reports on all methodological stages of its translation and cross-cultural adaptation process. They concluded that all methodological stages were conducted appropriately. No further adjustments were necessary.

## Discussion

Following international guidelines [39], we successfully translated and cross-culturally adapted the *Protocolo de Avaliação Miofuncional Orofacial MBGR* [26] from Brazilian Portuguese into English. The newly translated and cross-culturally adapted MBGR Protocol demonstrated excellent content and face validity. Below, we discuss the essential aspects of its translation and cross-cultural adaptation procedure.

Studies have examined the prevalence of OMDs in various population sub-groups [10, 16, 59–64] and pointed to a high prevalence of OMDs (38%) in the general population and an even higher one (81%) in children with speech and articulation problems. Studies have also indicated that OMDs can negatively impact orofacial postures and functions and significantly lower patients’ quality of life [6, 17, 34]. However, despite its relevance, scientific research on Orofacial Myology is basically conducted in Brazil and some European countries, such as Portugal and Spain [19]. Therefore, since most scientific research on Orofacial Myology is developed in Brazilian Portuguese, an increasing number of orofacial myofunctional assessment tools have been translated into English or other languages to allow for cross-national and cross-cultural studies and comparisons [2, 6, 7, 27–29].

In fact, to date, few orofacial myofunctional assessment tools developed in Brazilian Portuguese have been used in cross-national and cross-cultural studies [65–67]; nevertheless, this is likely to change given the increasing collaboration and connections between South American, European, and North American countries.

To translate and cross-culturally adapt a clinical assessment tool for use in a new country, culture, or language, we must follow a thorough method to reach semantic, idiomatic, experiential, and conceptual equivalences between its translated and original versions [39, 68]. Accordingly, researchers have developed thorough translation and cross-cultural adaptation guidelines to maximize the attainment of these equivalences and maintain the content validity of the clinical assessment tool across cultures [39, 68, 69]. Most Brazilian researchers, however, have not followed guidelines when translating and cross-culturally adapting orofacial myofunctional assessment tools [6, 7, 27–29]. Therefore, the core strength of this study is its rigorous translation and cross-cultural adaptation procedure, consisting of translations and synthesis of translations, back-translations, expert committee review, pretesting, and a process audit [39].

In the translation stage, both translators had English as their mother tongue and were fluent in Portuguese [39]. Also, the T1 translator had extensive experience translating health-related content and was aware of the purpose of the study [39]. The T2 translator, on the other hand, had little experience translating health-related content and was unaware of the purpose of the study [39]. As a result, T1 provided appropriate and reliable cross-cultural adaptations from a clinical perspective, and T2 reflected the colloquial English language.

During the synthesis of the translations, depending on the study’s target population, the translation units of T12 tend to remain mostly as in T1 or mostly as in T2 [39]. For example, in studies in which the target population consists of patients, it is common for the translation units of T12 to remain mostly as in T2 precisely because it reflects the colloquial English language. In this study, however, the target population consisted of healthcare professionals; so, considering their education and information levels, the translation units of T12 remained mostly as in T1 because it provided appropriate and reliable cross-cultural adaptations from a clinical perspective, especially regarding specialized terminology.

In the back-translation stage, both translators had Portuguese as their mother tongue and were fluent in English [39]. Beaton and colleagues also suggested that back-translators should have little experience translating health-related content and be unaware of the purpose of the study [39]. However, in this study, we worked with back-translators who had extensive experience translating health-related content and were aware of the purpose of the study. As researchers and professional translators, we know that problems may arise in the different stages of the translation and cross-cultural adaptation process. During the back-translation stage, for example, if the back-translators lack experience or are blinded to the purpose of the study, the result can be a lousy back-translation of a good translation [70, 71]. Therefore, the obvious solution to this problem is to work with informed and experienced translators.

It is fundamental for the expert committee to be staffed by bilingual, multidisciplinary professionals capable of revising all translation units of T1, T2, T12, BT1, and BT2 and resolving all discrepancies found between these translation units and their original versions [39]. Besides, in this stage, it is fundamental to utilize a structured technique to resolve these discrepancies [68]. So, in this study, considering the large number of translation units to be analyzed and evaluated and seeking to standardize how experts would analyze and evaluate all these translation units, we used the CVI [57]. The CVI allowed us to evaluate each translation unit independently by calculating its I-CVI, determine an overall average of the I-CVI scores for the assessment tool by calculating its S-CVI, and decide which translation units would be adjusted [57]. Additionally, it allowed us to confirm the content validity of the pre-final English version of the *Protocolo MBGR* with an S-CVI of 0.96 and 97% of the translation units with an I-CVI of 1.00 [57, 58].

In the fifth stage, to confirm whether the pre-final English version of the *Protocolo MBGR* worked in the target population’s linguistic-cultural setting and prove its face validity, we used it with 35 subjects. Since the assessment tool was developed for the exclusive use of healthcare professionals and patients do not have access to it, we worked with healthcare professionals: SLPs, in fact. Despite the number of subjects in the sample being in line with what is recommended by Beaton et al. [39], we acknowledge that we should have included professionals from different health areas in this stage because, in Brazil, SLPs are the most qualified professionals to work with Orofacial Myology [18]; in the United States, however, not only SLPs but also dentists, dental hygienists, physical therapists, and doctors, can work with Orofacial Myology if certified by AOMT or IAOM [17]. So, instead of working with SLPs who were ASHA members, we should have worked with professionals from different health areas who were certified by AOMT or IAOM. Nevertheless, our approach successfully captured relevant cross-cultural differences between Brazil and the United States and granted excellent cross-cultural adaptations. In addition, SLPs’ considerations revealed remarkable insights, especially regarding specialized terminology.

Furthermore, we divided the fifth stage into two phases: the pretest and the pilot test. The pretest consisted of a theoretical approach in which the SLPs focused on evaluating the linguistic content of the clinical assessment tool and identifying cross-cultural adaptation problems by reading its pre-final English version. While the pilot test consisted of a practical approach in which the SLPs got their hands on and identified cross-cultural adaptation problems that were only possible to notice when administering the clinical assessment tool. This practical approach was essential for the SLPs to develop the necessary psychomotor skills to administer the MBGR Protocol.

The MBGR Protocol is promising to enhance the uptake of studies in Orofacial Myology worldwide and support researchers and health professionals in assessing and diagnosing OMDs in children, adolescents, adults, and the elderly. Also, it may support evidence-based practice and assist in standardizing assessment and diagnostic criteria in Orofacial Myology. However, the MBGR Protocol should have its psychometric properties tested before being used in clinical practice or scientific research. Similar to its original Portuguese version, the MBGR Protocol can be adapted and validated for use in specific settings, including individuals with orofacial pain, temporomandibular disorders, speech disorders, and cleft lip and palate, for example. As already mentioned, the original Portuguese version of the *Protocolo MBGR* was validated for individuals with temporomandibular disorders [38], and this was due to its alignment with major consortia in temporomandibular disorders, including the International Network for Orofacial Pain and Related Disorders Methodology (INfORM). Therefore, future studies are needed, and collaborations among researchers from South and North American countries are encouraged to create an international network and advance with knowledge and skills in the Orofacial Myology discipline.

We understand that Brazilian technical and scientific knowledge in Orofacial Myology is of worldwide interest. So, considering English is the *lingua franca* of science, we translated and cross-culturally adapted the *Protocolo MBGR* into English. Still, as previously described in the guidelines [39], it may be necessary to cross-culturally adapt some items of the MBGR Protocol so that health professionals can use it in very specific settings. However, we believe that its specialized language will allow it to be used in these very specific settings without the need for additional cross-cultural adaptations.

## Supporting information

S1 Appendix MBGR protocol. (PDF)

S1 Dataset Data of the translation stage. (XLSX)

S2 Dataset Data of the synthesis of the translations stage. (XLSX)

S3 Dataset Data of the back-translation stage. (XLSX)

S4 Dataset Data of the expert committee stage. (XLSX)

S5 Dataset Data of the test of the pre-final version stage—pretest. (XLSX)

S6 Dataset Data of the test of the pre-final version stage—pilot test. (XLSX)

## References

[1] Machado PG, Mezzomo CL, Badaró AFV. Body posture and the stomatognathic functions in mouth breathing children: a literature review. Rev CEFAC. 2012;14(3):553–65. doi: 10.1590/S1516-18462012005000033

[2] Felício CM, Folha GA, Ferreira CLP, Paskay LC, Sforza C. Translation and cross-cultural adaptation of the protocol of orofacial myofunctional evaluation with scores for Italian Language. CoDAS. 2015;27(6):575–83. doi: 10.1590/2317-1782/20152015045 26691622

[3] Bigenzahn W, Fischman L, Mayrhofer-Krammel U. Myofunctional therapy in patients with orofacial dysfunctions affecting speech. Folia Phoniatr (Basel). 1992;44(5):238–44. doi: 10.1159/000266155 1490647

[4] Marchesan IQ. Lingual frenulum: classification and speech interference. Int J Orofacial Myology. 2004;30:31–8. 15832860

[5] Verrastro AP, Stefani FM, Rodrigues CR, Wanderley MT. Occlusal and orofacial myofunctional evaluation in children with anterior open bite before and after removal of pacifier sucking habit. Int J Orthod Milwaukee. 2007;18(3):19–25. 17958262

[6] Felício CM, Ferreira CLP. Protocol of orofacial myofunctional evaluation with scores. Int J Pediatr Otorhinolaryngol. 2008;72(3):367–75. doi: 10.1016/j.ijporl.2007.11.012 18187209

[7] Felício CM, Folha GA, Ferreira CLP, Medeiros APM. Expanded protocol of orofacial myofunctional evaluation with scores: Validity and reliability. Int J Pediatr Otorhinolaryngol. 2010;74(11):1230–9. doi: 10.1016/j.ijporl.2010.07.021 20800294

[8] Harari D, Redlich M, Miri S, Hamud T, Gross M. The effect of mouth breathing versus nasal breathing on dentofacial and craniofacial development in orthodontic patients. Laryngoscope. 2010;120(10):2089–93. doi: 10.1002/lary.20991 20824738

[9] Andrada e Silva MA, Marchesan IQ, Ferreira LP, Schmidt R, Ramires RR. Posture, lips and tongue tone and mobility of mouth breathing children. Rev CEFAC. 2012;14(5):853–60. doi: 10.1590/S1516-18462012005000002

[10] Leme MS, Barbosa TS, Gavião MBD. Assessment of orofacial functions in Brazilian children using the Nordic Orofacial Test-Screening (NOT-S). Rev Odonto Ciênc. 2012;27(2):108–14. doi: 10.1590/S1980-65232012000200003

[11] Hitos SF, Arakaki R, Solé D, Weckx LL. Oral breathing and speech disorders in children. J Pediatr (Rio J). 2013;89(4):361–5. doi: 10.1016/j.jped.2012.12.007 23809686

[12] Serel Arslan S, Demir N, Karaduman AA. Reliability and validity of a tool to measure the severity of tongue thrust in children: the Tongue Thrust Rating Scale. J Oral Rehabil. 2017;44(2):119–24. doi: 10.1111/joor.12471 27973693

[13] Rohrbach S, Buettner F, Pollex D, Mathmann P, Weinhold L, Schubert R, et al. Quantitative examination of isometric tongue protrusion forces in children with oro-facial dysfunctions or myofunctional disorders. J Oral Rehabil. 2018;45(3):228–34. doi: 10.1111/joor.12598 29230834

[14] Mariano NCR, Sano MN, Neppelenbroek KH, Almeida ALPF, Oliveira TM, Soares S. Orofacial Dysfunction In Cleft And Non-Cleft Patients Using Nordic Orofacial Test—A Screening Study. Braz Dent J. 2019;30(2):179–84. doi: 10.1590/0103-6440201902376 30970062

[15] Wuertz K, Glick A, Simmons J, Hansen-Kiss E. Pediatric Obstructive Sleep Medicine. In: Demerjian GG, Patel M, Chiappelli F, Barkhordarian A, editors. Dental Sleep Medicine: A Clinical Guide. Cham: Springer; 2022. p. 365–401.

[16] Wadsworth SD, Maul CA, Stevens EJ. The prevalence of orofacial myofunctional disorders among children identified with speech and language disorders in grades kindergarten through six. Int J Orofacial Myology. 1998;24:1–19. 10635163

[17] Mason RM. A retrospective and prospective view of orofacial myology. Int J Orofacial Myology. 2005;31:5–14. 16739708

[18] Susanibar F, Marchesan IQ, Santos R. World Orofacial Myofunctional Science Day. Rev CEFAC. 2015;17(5):1389–93. doi: 10.1590/1982-021620151752

[19] Moeller MR. The emerging area of orofacial myofunctional therapy: Efficacy of treatment in sleep disordered breathing bringing promise of a new field of medicine. Cranio. 2018;36(5):283–5. doi: 10.1080/08869634.2018.1512242 30257620

[20] Hanson ML, Mason RM. Orofacial Myology—International Perspectives. 2nd ed. Springfield: Charles C. Thomas Publisher; 2003.

[21] Moeller JL. Orofacial myofunctional therapy: why now? Cranio. 2012;30(4):235–6. doi: 10.1179/crn.2012.035 23156963

[22] Academy of Orofacial Myofunctional Therapy (AOMT). Frequently asked questions and answers in the area of Orofacial Myofunctional Therapy. 2014. [cited 2023 Oct 08]. Available from: https://aomtinfo.org/wp-content/uploads/2015/02/AOMT-brochure.pdf.

[23] Marchesan IQ, Krakauer LRH, Duarte LM, Bianchini EMG, Rahal A, Cattoni DM, et al. Documento Oficial 02/2002 do Comitê de Motricidade Oral (MO) da Sociedade Brasileira de Fonoaudiologia (SBFa). São Paulo: Art Color; 2002.

[24] American Speech-Language-Hearing Association. Evidence-Based Practice in Communication Disorders: An Introduction. 2005 [cited 2023 Jan 08]. Available from: https://www.asha.org/policy.

[25] Genaro KF, Berretin-Felix G, Rehder MIBC, Marchesan IQ. Orofacial myofunctional evaluation: MBGR Protocol. Rev CEFAC. 2009;11(2):237–55. doi: 10.1590/S1516-18462009000200009

[26] Berretin-Felix G, Genaro KF, Marchesan IQ. Protocolos de Avaliação da Motricidade Orofacial: Protocolo de Avaliação Miofuncional Orofacial MBGR. In: Silva HJ, Tessitore A, Motta AR, Cunha DA, Berretin-Felix G, Marchesan IQ, editors. Tratado de Motricidade Orofacial. 1 ed. São José dos Campos: Pulso; 2019. p. 255–72.

[27] Medeiros AMC, Nobre GRD, Barreto ÍDC, Jesus EMS, Folha GA, Matos ALS, et al. Expanded Protocol of Orofacial Myofunctional Evaluation with Scores for Nursing Infants (6–24 months) (OMES-E Infants). CoDAS. 2021;33(2):e20190219. doi: 10.1590/2317-1782/20202019219 34008774

[28] Medeiros AMC, Marchesan IQ, Genaro KF, Barreto ÍDC, Berretin-Felix G. MMBGR Protocol—Infants and Preschoolers: Instructive and Orofacial Myofunctional Clinical History. CoDAS. 2022;34(2):e20200324. doi: 10.1590/2317-1782/20212020324 35019077 PMC9851188

[29] Medeiros AMC, Marchesan IQ, Genaro KF, Barreto ÍDC, Berretin-Felix G. MMBGR Protocol—Infants and Preschoolers: Myofunctional Orofacial Clinic Examination. CoDAS. 2022;34(5):e20200325. doi: 10.1590/2317-1782/20212020325 Erratum in: CoDAS. 2023;35(1):e20220159. 35475847 PMC9886187

[30] Marchesan IQ, Berretin-Felix G, Genaro KF. MBGR protocol of orofacial myofunctional evaluation with scores. Int J Orofacial Myology. 2012;38:38–77. 23362752

[31] Paul-Brown D, Clausen R. Collaborative approach for identifying and treating speech, language, and orofacial myofunctional disorders. Alpha Omegan. 1999;92(2):39–44. 10850229

[32] Paskay LC. Instrumentation and measurement procedures in orofacial myology. Int J Orofacial Myology. 2006;32(1):37–57. doi: 10.52010/ijom.2006.32.1.4 17650767

[33] Badillo A. El portugués y el español en la ciencia: apuntes para un conocimiento diverso y accesible. Madrid: Organización de Estados Iberoamericanos para la Educación, la Ciencia y la Cultura (OEI) y Real Instituto Elcano; 2021.

[34] Leme MS, Barbosa TS, Gavião MBD. Relationship among oral habits, orofacial function and oral health-related quality of life in children. Braz Oral Res. 2013;27(3):272–8. doi: 10.1590/S1806-83242013000300006 23739786

[35] Pizolato RA, Freitas-Fernandes FS, Gavião MBD. Anxiety/depression and orofacial myofacial disorders as factors associated with TMD in children. Braz Oral Res. 2013;27(2):156–62. doi: 10.1590/s1806-83242013000100021 23538427

[36] Silva CM, Santos CA, Rezende NA. Orofacial myofunctional evaluation in individuals with neurofibromatosis type 1. Rev CEFAC. 2015;17(1):100–10. doi: 10.1590/1982-0216201515613

[37] Graziani AF, Fukushiro AP, Marchesan IQ, Berretin-Felix G, Genaro KF. Extension and validation of the protocol of orofacial myofunctional assessment for individuals with cleft lip and palate. CoDAS. 2019;31(1):e20180109. doi: 10.1590/2317-1782/20182018109 30843925

[38] Bueno MRS, Rosa RR, Genaro KF, Berretin-Felix G. Validation of the MBGR orofacial myofunctional assessment protocol for adults with temporomandibular disorders with disc displacement with reduction. CoDAS. 2020;32(4):e20190132. doi: 10.1590/2317-1782/20202019132 32321007

[39] Beaton DE, Bombardier C, Guillemin F, Ferraz MB. Guidelines for the process of cross-cultural adaptation of self-report measures. Spine. 2000;25(24):3186–91. doi: 10.1097/00007632-200012150-00014 11124735

[40] Janoca MÍG, Filho MOD, Vasconcelos FHP, Nascimento AS, Coury RMMMM, Barros CMB, et al. Oral health status and performance of oral functions in children and adolescents in the treatment for overweight or obesity. Open J Stomatol. 2013;3(9):59–64. doi: 10.4236/ojst.2013.39A009

[41] Melo DL, Santos RVM, Perilo TVC, Becker HMG, Motta AR. Mouth breathing evaluation: use of Glatzel mirror and peak nasal inspiratory flow. CoDAS. 2013;25(3):236–41. doi: 10.1590/s2317-17822013000300008 24408334

[42] Rosal AGC, Cordeiro AAA, Queiroga BAM. Phonological awareness and phonological development in children of public and private schools. Rev CEFAC. 2013;15(4):837–46. doi: 10.1590/S1516-18462013000400012

[43] Lima JAS, Luna AHB, Pessoa LSF, Alves GÂS. Functional gains measured by MBGR and impact on quality of life in subject submitted to orthognathic surgery: case report. Rev CEFAC. 2015;17(5):1722–30. doi: 10.1590/1982-021620151751015

[44] Migliorucci RR, Sovinski SRP, Passos DCBOF, Bucci AC, Salgado MH, Nary-Filho H, et al. Orofacial functions and quality of life in oral health in subjects with dentofacial deformity. CoDAS. 2015;27(3):255–9. doi: 10.1590/2317-1782/20152014162 26222942

[45] Prado DGA, Sovinski SRP, Nary-Filho H, Brasolotto AG, Berretin-Felix G. Oral motor control and orofacial functions in individuals with dentofacial deformity. Audiol Commun Res. 2015;20(1):76–83. doi: 10.1590/S2317-64312015000100001427

[46] Almeida LF, Lima MC, Macieira JC, César CPHAR, Baldrighi SEZM Speech Therapy in systemic sclerosis: a case report. Rev CEFAC. 2016;18(1):273–85. doi: 10.1590/1982-0216201618117715

[47] Sovinski SRP, Genaro KF, Migliorucci RR, Passos DCBOF, Berretin-Felix G. Aesthetic face evaluation in individuals with Dentofacial Deformities. Rev CEFAC. 2016;18(6):1348–58. doi: 10.1590/1982-0216201618622515

[48] Suzart DD, Carvalho ARR. Speech disorders related to alterations of the lingual frenulum in schoolchildren. Rev CEFAC. 2016;18(6):1332–9. doi: 10.1590/1982-0216201618621715

[49] Corrêa CC, José MR, Andrade EC, Feniman MR, Fukushiro AP, Berretin-Felix G, et al. Sleep quality and communication aspects in children. Int J Pediatr Otorhinolaryngol. 2017;100:57–61. doi: 10.1016/j.ijporl.2017.06.028 28802387

[50] Milanesi JM, Berwig LC, Marquezan M, Schuch LH, Moraes AB, Silva AMT, et al. Variables associated with mouth breathing diagnosis in children based on a multidisciplinary assessment. CoDAS. 2018;30(4):e20170071. doi: 10.1590/2317-1782/20182017071 29561967

[51] Minsky RC, Castilho T, Meira RRS, Bobbio TG, Schivinski CIS. Relationship between oral habits and spirometry maneuvers, in children. Rev CEFAC. 2018;20(1):37–43. doi: 10.1590/1982-0216201820110517

[52] Maia AV, Furlan RMMM, Moraes KO, Amaral MS, Medeiros AM, Motta AR. Tongue strength rehabilitation using biofeedback: a case report. CoDAS. 2019;31(5):e20180163. doi: 10.1590/2317-1782/20182018163 31664370

[53] Ferrer LJ, Tessitore A, Machado Júnior AJ, Sakano E. Clinical and surface electromyography evaluation pre and post orofacial myology therapy. Int J Orofacial Myology. 2020;46(1):5–12. doi: 10.52010/ijom.2020.46.1.1

[54] Melo PED, Bocato JR, Conti ACCF, Souza KRS, Fernandes TMF, Almeida MR, et al. Effects of orthodontic treatment with aligners and fixed appliances on speech. Angle Orthod. 2021;91(6):711–7. doi: 10.2319/110620-917.1 34037699 PMC8549558

[55] Scudine KG, Freitas CN, Moraes KSGN, Prado DA, Silveira PP, Castelo. Evaluation of masticatory behavior and taste sensitivity after pacifier removal in preschool children: a 1-year follow-up. Clin Oral Investig. 2022;26(5):4059–70. doi: 10.1007/s00784-022-04374-4 35147790

[56] Marchetti PT, Dalcin LM, Balen SA, Mezzomo CL. Temporal auditory processing and the distinctive features of children with phonological disorder. Rev CEFAC. 2022;24(3):e2022. doi: 10.1590/1982-0216/20222432022

[57] Polit DF, Beck CT. The content validity index: are you sure you know what’s being reported? Critique and recommendations. Res Nurs Health. 2006;29(5):489–97. doi: 10.1002/nur.20147 16977646

[58] Lynn MR. Determination and quantification of content validity. Nurs Res. 1986;35(6):382–5. 3640358

[59] Korbmacher H, Koch LE, Kahl-Nieke B. Orofacial myofunctional disorders in children with asymmetry of the posture and locomotion apparatus. Int J Orofacial Myology. 2005;31:26–38. 16739710

[60] Stahl F, Grabowski R, Gaebel M, Kundt G. Relationship between occlusal findings and orofacial myofunctional status in primary and mixed dentition. Part II: Prevalence of orofacial dysfunctions. J Orofac Orthop. 2007;68(2):74–90. doi: 10.1007/s00056-007-2606-9 17372707

[61] Ferreira CLP, Silva MAMR, Felício CM. Orofacial myofunctional disorder in subjects with temporomandibular disorder. Cranio. 2009;27(4):268–74. doi: 10.1179/crn.2009.038 19891261

[62] Hale ST, Kellum GD, Richardson JF, Messer SC, Gross AM, Sisakun S. Oral motor control, posturing, and myofunctional variables in 8-year-olds. J Speech Hear Res. 1992;35(6):1203–8. doi: 10.1044/jshr.3506.1203 1494265

[63] Folha GA, Valera FCP, Giglio LD, Trawizki LVV, Felício CM. Orofacial myofunctional evaluation with scores in subjects with obstructive sleep apnea. Sleep Med. 2013;14(1):e51. doi: 10.1016/j.sleep.2013.11.084

[64] Cavalcante-Leão BL, Todero SRB, Ferreira FM, Gavião MBD, Fraiz FC. Profile of orofacial dysfunction in Brazilian children using the Nordic Orofacial Test-Screening. Acta Odontol Scand. 2017;75(4):262–7. doi: 10.1080/00016357.2017.1290823 28358288

[65] Cañizares-Prado S, Molina-López J, Moya MT, Planells E. Oral Function and Eating Habit Problems in People with Down Syndrome. Int J Environ Res Public Health. 2022;19(5): 2616. doi: 10.3390/ijerph19052616 35270327 PMC8909609

[66] Begnoni G, Dellavia C, Pellegrini G, Scarponi L, Schindler A, Pizzorni N. The efficacy of myofunctional therapy in patients with atypical swallowing. Eur Arch Otorhinolaryngol. 2020;277(9):2501–11. doi: 10.1007/s00405-020-05994-w 32367149

[67] Mozzanica F, Pizzorni N, Scarponi L, Crimi G, Schindler A. Impact of Oral Myofunctional Therapy on Orofacial Myofunctional Status and Tongue Strength in Patients with Tongue Thrust. Folia Phoniatr Logop. 2021;73(5):413–21. doi: 10.1159/000510908 33113529

[68] Guillemin F, Bombardier C, Beaton DE. Cross-cultural adaptation of health-related quality of life measures: literature review and proposed guidelines. J Clin Epidemiol. 1993;46(12):1417–32. doi: 10.1016/0895-4356(93)90142-n 8263569

[69] Wild D, Grove A, Martin M, Eremenco S, McElroy S, Verjee-Lorenz A, et al. Principles of Good Practice for the Translation and Cultural Adaptation Process for Patient-Reported Outcomes (PRO) Measures: report of the ISPOR Task Force for Translation and Cultural Adaptation. Value Health. 2005;8(2):94–104. doi: 10.1111/j.1524-4733.2005.04054.x 15804318

[70] Bullinger M, Alonso J, Apolone G, Leplège A, Sullivan M, Wood-Dauphinee S, et al. Translating health status questionnaires and evaluating their quality: the IQOLA Project approach. International Quality of Life Assessment. J Clin Epidemiol. 1998;51(11):913–23. doi: 10.1016/s0895-4356(98)00082-1 9817108

[71] Peters M, Passchier J. Translating instruments for cross-cultural studies in headache research. Headache. 2006;46(1):82–91. doi: 10.1111/j.1526-4610.2006.00298.x 16412155

