# Supplementary material for: Translation and cross-cultural adaptation of the “Protocolo de Avaliação Miofuncional Orofacial MBGR” from Brazilian Portuguese into English
Source: PLoS One. 2023 Dec 4;18(12):e0295387. doi: 10.1371/journal.pone.0295387 (PMC10695393; doi:10.1371/journal.pone.0295387)
Supplement: S1 Appendix — (PDF) [file pone.0295387.s001.pdf]

MBGR PROTOCOL
OROFACIAL MYOFUNCTIONAL CLINICAL HISTORY
Marchesan IQ, Berretin-Felix G, Genaro KF, Rehder MIBC

Name: ____

Date of examination: ____ / ____ / ____ **Age:** ______________ **DoB:** ____ / ____ / ____

Relationship of person completing form: ❑ self ❑ parent ❑ caregiver ❑ other: __________________________

**Highest level of education:**					
**Employment:**	( ) employed	Job title:		( ) unemployed	( ) retired
**Previous work experience:**	( ) yes	( ) no	Describe:		
**Physical activity:**	( ) yes	( ) no	Describe:		

**Address 1:**			
**Address 2:**			
**City/State:**		**ZIP Code:**	
**Phone numbers:**	Home:	Work:	Mobile:
**Email:**			
**Father's name:**		**Mother's name:**	
**Siblings:**	( ) no	( ) yes	How many:

Who referred the patient to speech-language therapy? (name, specialty, and phone number)**:** ____________________

____
____
____

Main concern: ____

____
____
____
____
____
____
____

Other related concerns: (0) no (1) sometimes (2) yes

( ) lips	( ) tongue	( ) sucking	( ) chewing	( ) swallowing
( ) breathing	( ) speech	( ) lingual frenulum	( ) voice	( ) hearing
( ) learning	( ) facial appearance	( ) posture	( ) occlusion	( ) headache
( ) TMJ noise	( ) TMJ pain	( ) neck pain	( ) shoulder pain	
( ) difficulty in opening the mouth	( ) difficulty in moving the mandible side to side			

Family medical history

❑ no	❑ yes	Describe:

Complications

**During the mother's pregnancy:**	❑ no	❑ yes	Describe:
**At birth:**	❑ no	❑ yes	Describe:

Motor development

**Sitting:**	❑ normal	❑ delayed	Age:
**Walking:**	❑ normal	❑ delayed	Age:

Motor difficulty in: (0) no (1) sometimes (2) yes

( ) riding a bicycle	( ) running	( ) tying shoes	( ) getting dressed	( ) buttoning clothes
( ) others: ____________________				

Health problems

		**Describe**	**Treatment**	**Medication**
**Neurological:**	❑ no ❑ yes			
**Orthopedic:**	❑ no ❑ yes			
**Metabolic:**	❑ no ❑ yes			
**Digestive:**	❑ no ❑ yes			
**Hormonal:**	❑ no ❑ yes			

Other problems: ____

____
____

University of São Paulo
Bauru School of Dentistry
FAPESP
São Paulo Research Foundation

Copyright © 2020 by Giédre Berretin-Felix, Katia Flores Genaro, Irene Queiroz Marchesan, and Maria Inês Beltrati Cornacchioni Rehder.
All rights reserved. This clinical assessment tool or any portion thereof may be copied for clinical and academic purposes. Nevertheless, it may not be sold to a third party.

Breathing

			Frequency	Treatment	Medication
**Frequent colds*:**	❑ no	❑ yes			
**Throat problems:**	❑ no	❑ yes			
**Tonsillitis:**	❑ no	❑ yes			
**Halitosis:**	❑ no	❑ yes			
**Asthma:**	❑ no	❑ yes			
**Bronchitis:**	❑ no	❑ yes			
**Pneumonia:**	❑ no	❑ yes			
**Rhinitis:**	❑ no	❑ yes			
**Sinusitis:**	❑ no	❑ yes			
**Nasal obstruction:**	❑ no	❑ yes			
**Nasal itching:**	❑ no	❑ yes			
**Coryza** (nasal inflammation)**:**	❑ no	❑ yes			
**Repeated sneezing:**	❑ no	❑ yes			

*Frequent colds (infection of the upper airway): up to 5 years of age - over 12 episodes per year
between 6 and 12 years of age - over 6 episodes per year

Other problems: ____
____
____

Sleep

**Restless:**	❑ no	❑ yes	❑ sometimes
**Disrupted:**	❑ no	❑ yes	❑ sometimes
**Heavy snoring:**	❑ no	❑ yes	❑ sometimes
**Light snoring:**	❑ no	❑ yes	❑ sometimes
**Sialorrhea** (excessive salivation)**:**	❑ no	❑ yes	❑ sometimes
**Apnea:**	❑ no	❑ yes	❑ sometimes
**Water intake at night:**	❑ no	❑ yes	❑ sometimes
**Mouth open while sleeping:**	❑ no	❑ yes	❑ sometimes
**Dry mouth upon waking:**	❑ no	❑ yes	❑ sometimes
**Facial pain upon waking:**	❑ no	❑ yes	❑ sometimes
**Hand resting under the face:**	❑ no	❑ yes [ ] R [ ] L	❑ sometimes
**Posture:**	❑ side	❑ supine	❑ prone

Other problems: ____
____
____

Treatments

				Reason	Professional
**Speech-language therapy:**	❑ no	❑ in the past	❑ currently		
**Medical:**	❑ no	❑ in the past	❑ currently		
**Psychological:**	❑ no	❑ in the past	❑ currently		
**Physical therapy:**	❑ no	❑ in the past	❑ currently		
**Dental:**	❑ no	❑ in the past	❑ currently		
Procedure: [ ] extraction [ ] prosthesis [ ] implant [ ] fixed orthodontic appliances [ ] removable orthodontic appliances					
**Surgical:**	❑ no	❑ yes	Describe:	When:	

Other treatments: ____
____
____

Breast or bottle feeding

**Breast:**	❑ no	❑ yes	For how long:
**Bottle:**	❑ no	❑ yes	For how long:

Diet - challenges in introducing:

**Utensils:**	❑ no	❑ yes	Describe:
**Flavors:**	❑ no	❑ yes	Describe:
**Consistencies:**	❑ no	❑ yes	Describe:

Copyright © 2020 by Giédre Berretin-Felix, Katia Flores Genaro, Irene Queiroz Marchesan, and Maria Inês Beltrati Cornacchioni Rehder.
All rights reserved. This clinical assessment tool or any portion thereof may be copied for clinical and academic purposes. Nevertheless, it may not be sold to a third party.

Current diet

				Describe
**Fruits:**	❑ no	❑ yes	❑ sometimes	
**Greens:**	❑ no	❑ yes	❑ sometimes	
**Vegetables:**	❑ no	❑ yes	❑ sometimes	
**Cereals** (rice, corns, oats)**:**	❑ no	❑ yes	❑ sometimes	
**Grains** (beans, lentils, peas)**:**	❑ no	❑ yes	❑ sometimes	
**Meats:**	❑ no	❑ yes	❑ sometimes	
**Dairies:**	❑ no	❑ yes	❑ sometimes	
**Sweets:**	❑ no	❑ yes	❑ sometimes	

In general, the patient's diet mostly consists of:

❑ liquids	❑ purees	❑ solids

Most of the time, how and where does the patient eat:

**While reading:**	❑ at the table	❑ on the sofa	❑ on the floor	❑ in bed
**While watching TV:**	❑ at the table	❑ on the sofa	❑ on the floor	❑ in bed
**While doing homework:**	❑ at the table	❑ on the sofa	❑ on the floor	❑ in bed
**While using the computer:**	❑ at the table	❑ on the sofa	❑ on the floor	❑ in bed
**Without any other activity:**	❑ at the table	❑ on the sofa	❑ on the floor	❑ in bed

Chewing

**Side:**	❑ bilateral	❑ unilateral [ ] R [ ] L	
**Lips:**	❑ closed	❑ parted	❑ open
**Noise:**	❑ no	❑ yes	❑ sometimes
**Liquid intake during meals:**	❑ no	❑ yes	❑ sometimes
**Pain or discomfort while chewing:**	❑ no	❑ yes [ ] R [ ] L	❑ sometimes
**TMJ noise:**	❑ no	❑ yes [ ] R [ ] L	❑ sometimes
**Difficulty in chewing:**	❑ no	❑ yes Describe:	
**Spillage of food while chewing:**	❑ no	❑ yes	

Other problems: ____
____
____

Chewing of food

❑ normal	❑ reduced	❑ prolonged

Chewing speed

	similar	faster	slower
**Compared to family:**	❑	❑	❑
**Compared to friends:**	❑	❑	❑

Chewing capacity (patient's level of satisfaction concerning their chewing)

❑ great	❑ good	❑ regular	❑ bad	❑ terrible

Swallowing

**Difficulty in swallowing:**	❑ no	❑ yes	❑ sometimes
**Noise:**	❑ no	❑ yes	❑ sometimes
**Choking:**	❑ no	❑ yes	❑ sometimes
**Odynophagia** (pain when swallowing)**:**	❑ no	❑ yes	❑ sometimes
**Nasal reflux:**	❑ no	❑ yes	❑ sometimes
**Anterior escape:**	❑ no	❑ yes	❑ sometimes
**Phlegm:**	❑ no	❑ yes	❑ sometimes
**Coughing:**	❑ no	❑ yes	❑ sometimes
**Residues after swallowing:**	❑ no	❑ yes	❑ sometimes

Other problems: ____
____
____

Copyright © 2020 by Giédre Berretin-Felix, Katia Flores Genaro, Irene Queiroz Marchesan, and Maria Inês Beltrati Cornacchioni Rehder.
All rights reserved. This clinical assessment tool or any portion thereof may be copied for clinical and academic purposes. Nevertheless, it may not be sold to a third party.

Oral habits

**Pacifier sucking:**	❑ no	❑ yes	For how long:	[ ] conventional [ ] orthodontic
**Finger or thumb sucking:**	❑ no	❑ yes	For how long:	
**Tongue sucking:**	❑ no	❑ yes	For how long:	
**Lip moistening:**	❑ no	❑ yes	Age:	
**Smoking:**	❑ no	❑ yes	How many cigarettes per day:	

Other habits: ____

____

____

Biting habits

**Bruxism** (teeth grinding)**:**	❑ no	❑ yes [ ] during the day [ ] during the night	
**Teeth clenching:**	❑ no	❑ yes	When:
**Onychophagy** (nail biting)**:**	❑ no	❑ yes	When:
**Biting of oral mucosa:**	❑ no	❑ yes	When:
**Biting of objects:**	❑ no	❑ yes	When: Describe:

Other habits: ____

____

____

Posture habits

**Lower lip interposition:**	❑ no	❑ yes	
**Mandible protrusion:**	❑ no	❑ yes	
**Mandible resting on hand:**	❑ no	❑ yes [ ] R [ ] L	
**Head resting on hand:**	❑ no	❑ yes [ ] R [ ] L	
**Excessive use of computer:**	❑ no	❑ yes	Posture:
**Excessive use of mobile phone:**	❑ no	❑ yes	Posture:

Other habits: ____

____

____

Speech

**Omission:**	❑ no	❑ yes	❑ sometimes	
**Substitution:**	❑ no	❑ yes	❑ sometimes	
**Impaired intelligibility:**	❑ no	❑ yes	❑ sometimes	
**Impaired intelligibility on the phone:**	❑ no	❑ yes	❑ sometimes	
**Excessive salivation:**	❑ no	❑ yes	❑ sometimes	
**Decreased range of mandibular movements:**	❑ no	❑ yes	❑ sometimes	
**Tongue interposition:**	❑ no	❑ yes [ ] anterior [ ] lateral		Describe:

Other problems: ____

____

____

Communication

**Delayed intentional communication:**	❑ no	❑ yes
**Delayed babble:**	❑ no	❑ yes
**Delayed onset of speech:**	❑ no	❑ yes
**Delayed onset of language:**	❑ no	❑ yes
**Difficulty in comprehending:**	❑ no	❑ yes

Other problems: ____

____

____

Hearing

**Hypoacusis** (hearing loss)**:**	❑ no	❑ yes [ ] R [ ] L	❑ sometimes
**Otitis** (ear infection)**:**	❑ no	❑ yes [ ] R [ ] L	❑ sometimes
**Tinnitus:**	❑ no	❑ yes [ ] R [ ] L	❑ sometimes
**Otalgia** (earache)**:**	❑ no	❑ yes [ ] R [ ] L	❑ sometimes
**Dizziness or vertigo:**	❑ no	❑ yes	❑ sometimes
**Previous audiological assessment:**	❑ no	❑ yes When:	

Other problems: ____

____

____

Copyright © 2020 by Giédre Berretin-Felix, Katia Flores Genaro, Irene Queiroz Marchesan, and Maria Inês Beltrati Cornacchioni Rehder.
All rights reserved. This clinical assessment tool or any portion thereof may be copied for clinical and academic purposes. Nevertheless, it may not be sold to a third party.

Voice

**Hoarseness:**	❑ no	❑ yes	❑ sometimes
**Hypophonia:**	❑ no	❑ yes	❑ sometimes
**Hypernasality:**	❑ no	❑ yes	❑ sometimes
**Hyponasality:**	❑ no	❑ yes	❑ sometimes
**Aphonia:**	❑ no	❑ yes	❑ sometimes
**Vocal abuse:**	❑ no	❑ yes	❑ sometimes
**Odynophonia** (pain when speaking)**:**	❑ no	❑ yes	❑ sometimes
**Burning sensation when speaking:**	❑ no	❑ yes	❑ sometimes

Other problems: ____
____
____

Education

**Learning difficulty:**	❑ no	❑ yes	Describe:
**Lack of attention or concentration:**	❑ no	❑ yes	❑ sometimes
**Memory difficulty:**	❑ no	❑ yes	
**Grade retentions:**	❑ no	❑ yes	How many:
**Difficulty with relationships:**	❑ no	❑ yes	
**Handedness:**	❑ right-handed	❑ left-handed	❑ ambidextrous

Other difficulties: ____
____
____

Additional information: ____
____
____
____
____
____
____
____
____
____
____
____
____
____
____
____
____
____
____
____
____
____
____
____
____
____
____
____
____
____
____

Speech-Language Pathologist: ____

Copyright © 2020 by Giédre Berretin-Felix, Katia Flores Genaro, Irene Queiroz Marchesan, and Maria Inês Beltrati Cornacchioni Rehder.
All rights reserved. This clinical assessment tool or any portion thereof may be copied for clinical and academic purposes. Nevertheless, it may not be sold to a third party.

Name: ____

Date of examination: _____ / _____ / _____ **Age:** _____________________ **DoB:** _____ / _____ / _____

Weight: ________ lb. **Height:** _______ ft. _______ in.

1. BODY POSTURE (observe the patient standing barefoot)

Head

(flexion and extension = yes) (rotation = no) (inclination = maybe)

**Frontal:**	( ) normal	( ) rotation R	( ) rotation L	( ) inclination R	( ) inclination L
**Lateral:**	( ) normal	( ) forward head posture	( ) flexion	( ) extension	

Shoulders

**Frontal:**	( ) normal	( ) elevated R	( ) elevated L
**Lateral:**	( ) normal	( ) rounded shoulders	

Observation: ____

2. MEASUREMENTS OF THE FACE, MANDIBULAR MOVEMENTS, AND OCCLUSION

Face (using a caliper, take each measurement three times, and calculate the average) (the patient must keep lips closed)

	**1st meas.** (in.)	**2nd meas.** (in.)	**3rd meas.** (in.)	**average** (in.)
**Middle third of the face** (from glabella to subnasal)				
**Lower third of the face** (from subnasal to gnathion)				
**Facial length - L** (from glabella to gnathion) (sum of the middle and lower thirds)				
**Facial width - W** (prominences of the zygomatic arches) (this measurement will be more accurate if measured with a spreading caliper with an extension of at least four inches)				
**Outer corner of the right eye to the right commissure of the lips**				
**Outer corner of the left eye to the left commissure of the lips**				
**Upper lip** (from subnasal to the lowermost point of the upper lip)				
**Lower lip** (from the uppermost point of the lower lip to gnathion)				

Mandibular movements and occlusion (using a caliper and a pencil, take each measurement three times, and calculate the average)

	**1st meas.** (in.)	**2nd meas.** (in.)	**3rd meas.** (in.)	**average** (in.)
**Overbite - OB** (with the teeth in occlusion, mark the incisal edge of the upper incisors on the vestibular surface of the lower incisors, and measure the distance from the marking to the incisal edge of the lower incisors; in the open bite, measure the distance between the incisal edges of the upper and lower incisors on the vertical plane, and the result will be negative)				
**Overjet - OJ** (measure the distance between the incisal edges of the upper and lower incisors on the horizontal plane)				
**Right lateral mandibular movement** (mark the dental midline of the upper arch on the lower arch, move the mandible to the right, and measure the distance between the marking and the upper midline)				
**Left lateral mandibular movement** (mark the dental midline of the upper arch on the lower arch, move the mandible to the left, and measure the distance between the marking and the upper midline)				
**Maximal interincisal distance - MID** (from the central or lateral upper incisor to the lower incisor with the maximum mouth opening)				
Mouth opening (MID + OB)				
(MID) with tongue tip touching the incisive papilla (MIDTIP)				
Calculate: (MIDTIP) x 100 / (MID)				

3. EXTRAORAL EXAMINATION [ ] Sum of the scores for face, lips, and masseter (best result = 0; worst result = 30)

Face [ ] Sum of the scores for frontal and lateral views (best result = 0; worst result = 15) (observe the patient standing barefoot)

Frontal view [ ] Sum of the scores for numerical and subjective facial analyses (best result = 0; worst result = 13)

Numerical facial analysis [ ] Sum of all scores (best result = 0; worst result = 3)

**Facial type**			
Compare length (L) with width (W):	(0) similar (L similar to W)	(1) long (L > W)	(1) short (L < W)
**Facial proportion**			
Compare the middle with the lower third:	(0) similar	(1) lower third larger	(1) lower third smaller
Compare R & L distances from the outer corner of the eye to the commissure of the lips:		(0) similar	(1) asymmetrical

Copyright © 2020 by Giédre Berretin-Felix, Katia Flores Genaro, Irene Queiroz Marchesan, and Maria Inês Beltrati Cornacchioni Rehder.
All rights reserved. This clinical assessment tool or any portion thereof may be copied for clinical and academic purposes. Nevertheless, it may not be sold to a third party.

Subjective facial analysis [ ] Sum of all scores (best result = 0; worst result = 10)

	Symmetrical	Asymmetrical	Describe
**Infraorbital plane**	(0)	(1)	
**Zygomatic arches**	(0)	(1)	
**Nasal alae**	(0)	(1)	
**Cheeks**	(0)	(1)	
**Nasolabial sulci**	(0)	(1)	
**Upper lip**	(0)	(1)	
**Lower lip**	(0)	(1)	
**Commissure of the lips**	(0)	(1)	
**Chin**	(0)	(1)	
**Mandible** (body and ramus)	(0)	(1)	

Lateral view [ ] Sum of all scores (best result = 0; worst result = 2)

**Pattern:**	(0) pattern I (straight)	(1) pattern II (convex)	(1) pattern III (concave)
**Nasolabial angle:**	(0) near 90º - 110°	(1) acute (< 90º)	(1) obtuse (> 110º)

Observation: ____

Lips [ ] Sum of all scores (best result = 0; worst result = 13)

**Resting posture:**		(0) closed	(1) closed with tension	(2) sometimes open/closed
		(2) parted	(2) closed with dental contact	(3) open
**Shape:**	Upper:	(0) normal (1st cupid's bow)	(1) gull wing (1st and 2nd cupid's bows)	
	Lower:	(0) normal	(1) with mild eversion	(2) with severe eversion
**Upper lip length:**		(0) covers ⅔ of incisors	(1) covers more than ⅔ of incisors	(1) covers less than ⅔ of incisors
		(0) ½ of lower's measurement	(1) more than ½ of lower's measurement	(2) less than ½ of lower's measurement
**External mucosa:**	(0) normal	(1) moist	(1) dry	(2) injured

Observation: ____

Masseter [ ] Sum of all scores (best result = 0; worst result = 2) (examine by visual observation and palpation)

**At rest:**	(0) relaxed	(1) contracted (clenched teeth)	
**Muscle recruitment in isometric contraction:**	(0) simultaneous	(1) first R side	(1) first L side

Observation: ____

4. INTRAORAL EXAMINATION [ ] Sum of the scores for lips, cheeks, tongue, palate, palatine tonsils, teeth, and occlusion (best result = 0; worst result = 61)

Lips [ ] Sum of all scores (best result = 0; worst result = 5)

**Buccal mucosa:**	(0) normal	(1) marked by teeth	(2) injured
**Upper frenulum:**	Attachment to the alveolar ridge:	(0) adequate	(1) low
	Thickness:	(0) adequate	(1) altered (describe):

Observation: ____

Cheeks [ ] Sum of all scores (best result = 0; worst result = 8)

**Mucosa:**	(0) normal	(1) marked by teeth or orthodontic appliances R	(1) linea alba R (white line on the buccal mucosa)	(2) injured R
		(1) marked by teeth or orthodontic appliances L	(1) linea alba L (white line on the buccal mucosa)	(2) injured L

Observation: ____

Tongue [ ] Sum of all scores (best result = 0; worst result = 20)

**Resting posture:** ❑ not observable	(1) on the floor of the mouth	(1) low tip and high back	(1) interdental:	
**Symmetry:** (0) yes	(1) no (describe):			
**Width:** (0) adequate	(1) reduced	(2) increased		
**Length:** (0) adequate	(1) increased			
**Mucosa:** (0) normal	(1) geographical	(1) fissured	(2) injured (region):	
(1) marked by teeth (region):	(1) marked by orthodontic appliances (region):			
**Frenulum:** Attachment to the floor of the mouth:	(0) between the sublingual folds	(1) on the alveolar crest		
Attachment to the tongue:	(0) on the middle part	(1) before the middle part	(2) at the tongue tip	
Shape of tongue tip while elevating the tongue:	(0) rounded	(1) square or rectangular	(1) slight crevice at the tongue tip	
		(2) heart-shaped	(3) does not elevate	
Other characteristics: (0) none	(1) submucosal	(1) thick	(1) fibrous	

Observation: ____

University of São Paulo
Bauru School of Dentistry
FAPESP
São Paulo Research Foundation

Copyright © 2020 by Giédre Berretin-Felix, Katia Flores Genaro, Irene Queiroz Marchesan, and Maria Inês Beltrati Cornacchioni Rehder.
All rights reserved. This clinical assessment tool or any portion thereof may be copied for clinical and academic purposes. Nevertheless, it may not be sold to a third party.

Palate [ ] Sum of all scores (best result = 0; worst result = 8)

**Hard palate:**	Depth: (0) adequate	(1) reduced (low)	(2) increased (high)
	Width: (0) adequate	(1) increased (wide)	(2) reduced (narrow)
**Soft palate:**	Symmetry: (0) symmetrical	(1) asymmetrical (describe):	
	Length: (0) adequate	(1) long	(2) short
**Uvula:** (0) adequate	(1) altered (describe):		

Observation: ____

Palatine tonsils [ ] Sum of all scores (best result = 0; worst result = 4)

**Presence:**	❑ present	❑ removed	❑ not observable
**Size:**	(0) adequate	(1) hypertrophy R	(1) hypertrophy L
**Color:**	(0) adequate	(1) hyperemia R	(1) hyperemia L

Observation: ____

Teeth [ ] Sum of all scores (best result = 0; worst result = 5)

**Dentition:** ❑ deciduous	❑ mixed	❑ permanent		
**Number of teeth:** upper R:	upper L:	lower R:	lower L:	
**Dental defects:** (0) absent	(1) present (describe):			
**Oral health:** Teeth:	(0) good	(1) regular	(2) poor	
Gums:	(0) good	(1) regular	(2) poor	
**Use of prosthesis:** ❑ no	❑ removable	❑ fixed	❑ partial	❑ full

Observation: ____

Occlusion [ ] Sum of all scores (best result = 0; worst result = 11)

**Midline:** (0) adequate	(1) deviated to R	(1) deviated to L		
**Angle's classification:** R side:	(0) Class I	(1) Class II div. 1st	(1) Class II div. 2nd	(1) Class III
L side:	(0) Class I	(1) Class II div. 1st	(1) Class II div. 2nd	(1) Class III
**Lateral guidance:** (0) present	(1) absent R	(1) absent L		
**Horizontal relationship:**	(0) adequate [OJ between 0.03 and 0.11 in.]	(1) excessive overjet [OJ > 0.11 in.]		
	(1) top bite [OJ = 0 in.]	(1) anterior crossbite [OJ < 0 in.]		
**Vertical relationship:**	(0) adequate [OB between 0.03 and 0.11 in.]	(1) excessive overbite [OB > 0.11 in.]	(1) posterior open bite R	
	(1) top bite [OB = 0 in.]	(1) anterior open bite [OB < 0 in.]	(1) posterior open bite L	
**Transversal relationship:** (0) adequate	(1) posterior crossbite R	(1) posterior crossbite L		
**Use of orthodontic appliances:** ❑ no	❑ removable	❑ fixed		

Observation: ____

5. MOBILITY [ ] Sum of the scores for lips, tongue, soft palate, and mandible (best result = 0; worst result = 68)

Lips [ ] Sum of all scores (best result = 0; worst result = 27) (*the patient must keep teeth occluded)

	Adequate	Slight alteration	Severe alteration	Absent
**Protrusion of closed lips*:**	(0)	(1)	(2)	(3)
**Retraction of closed lips*:**	(0)	(1)	(2)	(3)
**Protrusion of open lips*:**	(0)	(1)	(2)	(3)
**Retraction of open lips*:**	(0)	(1)	(2)	(3)
**Protrusion of closed lips to the R*:**	(0)	(1)	(2)	(3)
**Protrusion of closed lips to the L*:**	(0)	(1)	(2)	(3)
**Protrusion of closed lips and alternation to the L and R*:**	(0)	(1)	(2)	(3)
**Popping of protruded lips:**	(0)	(1)	(2)	(3)
**Popping of retracted lips:**	(0)	(1)	(2)	(3)

Observation: ____

Tongue [ ] Sum of all scores (best result = 0; worst result = 24)

	Adequate	Slight alteration	Severe alteration	Absent
**Tongue protrusion:**	(0)	(1)	(2)	(3)
**Touch of tongue tip sequentially at R and L commissures and U and L lips:**	(0)	(1)	(2)	(3)
**Touch of tongue tip on the incisive papilla:**	(0)	(1)	(2)	(3)
**Touch of tongue tip on the R cheek:**	(0)	(1)	(2)	(3)
**Touch of tongue tip on the L cheek:**	(0)	(1)	(2)	(3)
**Click of tongue tip:**	(0)	(1)	(2)	(3)
**Sucking of tongue against the palate:**	(0)	(1)	(2)	(3)
**Tongue trill:**	(0)	(1)	(2)	(3)

Observation: ____

Copyright © 2020 by Giédre Berretin-Felix, Katia Flores Genaro, Irene Queiroz Marchesan, and Maria Inês Beltrati Cornacchioni Rehder.
All rights reserved. This clinical assessment tool or any portion thereof may be copied for clinical and academic purposes. Nevertheless, it may not be sold to a third party.

Soft palate [ ] Sum of all scores (best result = 0; worst result = 4)

	Adequate		Reduced		Absent	
**Prolonged utterance of the vowel /a/ repeatedly:**	(0) R	(0) L	(1) R	(1) L	(2) R	(2) L

Observation: ____

Mandible [ ] Sum of all scores (best result = 0; worst result = 13)

	Adequate	Reduced	Increased	Unable to perform	With deviation		With noise	With pain
**Mouth opening:**	(0)	(1)	(1)	(2)	(1) R	(1) L	(1)	(1)
Expected values: child = 1.37 in. to 1.96 in. and adult = 1.57 in. to 2.16 in.								
**Mouth closure:**	(0)	-	-	-	(1) R	(1) L	(1)	(1)
**Lateral movement to the R:**	(0)	(1)	(1)	(2)	-		(1)	(1)
**Lateral movement to the L:**	(0)	(1)	(1)	(2)	-		(1)	(1)
Expected values: child = 0.23 in. to 0.39 in. and adult = 0.31 in. to 0.47 in.								

Observation: ____

6. SENSITIVITY [ ] Sum of the scores for tactile and pain on palpation (best result = 0; worst result = 65)

Tactile [ ] Sum of all scores (best result = 0; worst result = 55) (use the esthesiometer)

		Green filament	Blue filament	Violet filament	Dark red filament	Orange filament	Magenta filament
**Lips** (center)**:**	Upper:	(0)	(1)	(2)	(3)	(4)	(5)
	Lower:	(0)	(1)	(2)	(3)	(4)	(5)
**Tongue:**	Anterior region:	(0)	(1)	(2)	(3)	(4)	(5)
	Posterior region:	(0)	(1)	(2)	(3)	(4)	(5)
**Incisive papilla:**		(0)	(1)	(2)	(3)	(4)	(5)
**Chin** (center)**:**		(0)	(1)	(2)	(3)	(4)	(5)
**Cheeks** (center)**:**	Internal R:	(0)	(1)	(2)	(3)	(4)	(5)
	Internal L:	(0)	(1)	(2)	(3)	(4)	(5)
	External R:	(0)	(1)	(2)	(3)	(4)	(5)
	External L:	(0)	(1)	(2)	(3)	(4)	(5)
**Other regions:**		(0)	(1)	(2)	(3)	(4)	(5)

Pain on palpation [ ] Sum of all scores (best result = 0; worst result = 10)

	Absent		Present	
**Anterior temporalis:**	(0) R	(0) L	(1) R	(1) L
**Superficial masseter:**	(0) R	(0) L	(1) R	(1) L
**Trapezius:**	(0) R	(0) L	(1) R	(1) L
**Sternocleidomastoid:**	(0) R	(0) L	(1) R	(1) L
**TMJ:**	(0) R	(0) L	(1) R	(1) L

7. TONE [ ] Sum of all scores (best result = 0; worst result = 6) (examine by visual observation and palpation)

	Normal	Reduced	Increased
**Upper lip:**	(0)	(1)	(1)
**Lower lip:**	(0)	(1)	(1)
**Chin:**	(0)	(1)	(1)
**Tongue:**	(0)	(1)	(1)
**Right cheek:**	(0)	(1)	(1)
**Left cheek:**	(0)	(1)	(1)

Observation: ____

8. OROFACIAL FUNCTIONS [ ] Sum of the scores for breathing, chewing, swallowing, and speech (best result = 0; worst result = 123)

Breathing [ ] Sum of all scores (best result = 0; worst result = 5)

If altered, the source is [ ] functional [ ] structural [ ] other: ______________________________

**Type:** (0) middle/low	(1) middle/high	(1) other (describe):		
**Mode:** (0) nasal	(1) oronasal	(2) oral		
**Nasal breathing:**	(0) 2 minutes or more	(1) between 1 and 2 minutes	(2) less than 1 minute	
**Nasal flow:** (use a mirror) Upon arrival:	❑ similar between nostrils	❑ slight asymmetry	❑ moderate asymmetry	❑ severe asymmetry
After cleaning:	❑ similar between nostrils	❑ slight asymmetry	❑ moderate asymmetry	❑ severe asymmetry

Observation: ____

Copyright © 2020 by Giédre Berretin-Felix, Katia Flores Genaro, Irene Queiroz Marchesan, and Maria Inês Beltrati Cornacchioni Rehder.
All rights reserved. This clinical assessment tool or any portion thereof may be copied for clinical and academic purposes. Nevertheless, it may not be sold to a third party.

Chewing [ ] Sum of all scores (best result = 0; worst result = 10)

If altered, the source is [ ] functional [ ] structural [ ] TMD [ ] other: ___________________

Habitual chewing (always use the same food and analyze by video)

**Incision:**	(0) anterior	(1) lateral	(1) other:
**Chewing:**	(0) posterior teeth<br>(0) efficient	(1) anterior teeth<br>(1) inefficient	(1) with the tongue (tongue mashing)

**Number of cycles:**	**1st bite**	**2nd bite**	**3rd bite**	**Total**	(%)
**Right:**	________	________	________	________	(%)
**Left:**	________	________	________	________	(%)
**Right and left:**	________	________	________	________	(%)
**Total:**	________	________	________	________	(100%)

**Chewing pattern:**	(0) alternating unilateral or bilateral (50% - 65%)<br>(0) preferential unilateral (66% - 75%):		(1) simultaneous bilateral (> 65%)<br>(2) chronic unilateral (> 75%):
**Lip closure:**	(0) systematic	(1) unsystematic	(2) absent
**Noisy chewing:**	(0) no	(1) yes	
**Unexpected muscular contractions:**		(0) absent	(1) present (describe):
**Rhythm:** (0) adequate	(1) slow	(1) fast	

Observation: ___________________

Patient responses

**Preferred side to chew:** ❑ right and left	❑ right	❑ left	❑ does not know
**Chewing pain:** ❑ absent	❑ right	❑ left	
**TMJ noise:** ❑ absent	❑ right	❑ left	

Observation: ___________________

Swallowing [ ] Sum of the scores for the three swallowing tests (best result = 0; worst result = 36)

If altered, the source is [ ] functional [ ] structural [ ] other: ___________________

Habitual swallowing of solids [ ] Sum of all scores (best result = 0; worst result = 15)

**Lip posture:** (0) closed	(1) partially closed	(1) lower lip touching the upper teeth	(2) open	
**Tongue posture:** ❑ not observable	(0) behind the teeth	(1) against the teeth	(2) between the teeth	
**Containment of food:**	(0) adequate	(1) partial	(2) inadequate	
**Orbicularis contraction:**	(0) adequate	(1) little	(2) accentuated	
**Mentalis contraction:**	(0) absent	(1) little	(2) accentuated	
**Head movement:**	(0) absent	(1) present		
**Noise:** (0) absent	(1) present			
**Coordination:** (0) normal	(1) choking	(1) coughing		
**Residues after swallowing:**	(0) absent	(1) present		

Observation: ___________________

Habitual swallowing of liquids (water) **[ ]** Sum of all scores (best result = 0; worst result = 8)

**Tongue posture:** ❑ not observable	(0) behind the teeth	(1) against the teeth	(2) between the teeth
**Containment of liquid:** (0) adequate	(1) inadequate		
**Amount of liquid:** (0) adequate	(1) increased	(1) reduced	
**Noise:** (0) absent	(1) present		
**Rhythm:** (0) sequential	(1) sip by sip		
**Coordination:** (0) normal	(1) choking	(1) coughing	

Observation: ___________________

Guided swallowing (liquid - water) **[ ]** Sum of all scores (best result = 0; worst result = 13) (request the patient to hold water in their mouth and swallow it only after the evaluator's command)

**Lip posture:** (0) closed	(1) partially closed	(1) lower lip touching the upper teeth	(2) open
**Tongue posture:**	(0) behind the teeth	(1) against the teeth	(2) between the teeth
**Containment of liquid:**	(0) adequate	(1) partial	(2) inadequate
**Orbicularis contraction:**	(0) adequate	(1) little	(2) accentuated
**Mentalis contraction:**	(0) absent	(1) little	(2) accentuated
**Head movement:**	(0) absent	(1) present	
**Noise:** (0) absent	(1) present		
**Coordination:** (0) normal	(1) choking	(1) coughing	

Observation: ___________________

Copyright © 2020 by Giédre Berretin-Felix, Katia Flores Genaro, Irene Queiroz Marchesan, and Maria Inês Beltrati Cornacchioni Rehder.
All rights reserved. This clinical assessment tool or any portion thereof may be copied for clinical and academic purposes. Nevertheless, it may not be sold to a third party.

Patient responses

**Difficulty in swallowing:** ❑ no	❑ yes (describe):		
**Tongue position:** ❑ behind the upper teeth	❑ behind the lower teeth	❑ between the teeth	❑ does not know

Observation: ____

Speech [   ] Sum of the scores for the four speech tests (best result = 0; worst result = 72)
Phonetic/phonological aspect [   ] Sum of all scores (best result = 0; worst result = 30)
Characteristics: ❑ Phonological ❑ Phonetic/Phonological
❑ Phonetic: [ ] functional [ ] structural [ ] TMD [ ] neuromuscular [ ] other: ________________________
(0) absence (1) unsystematic presence (2) systematic presence

**Spontaneous speech [ ]** (best result = 0; worst result = 8)<br>“say your name and age,” “talk about your studies or work,” “talk about a trip you took”<br>[ ] Omission* [ ] Substitution* [ ] Distortion [ ] Change in place of articulation [ ] Tongue projection		
**Automatic speech [ ]** (best result = 0; worst result = 8)<br>“count from 1 to 20,” “list the days of the week,” “name the months of the year”<br>[ ] Omission* [ ] Substitution* [ ] Distortion [ ] Change in place of articulation [ ] Tongue projection		
**Naming of pictures or reading of words [ ]** (best result = 0; worst result = 8)<br>(use phonetically balanced pictures and their corresponding words)<br>[ ] Omission* [ ] Substitution* [ ] Distortion [ ] Change in place of articulation [ ] Tongue projection		
*If expected for the age, score zero (0)<br>Phones/phonemes and characteristics:		
In the case of omission and/or substitution:	❑ expected for the patient’s age	❑ not expected for the patient’s age
In the case of change in place of articulation:	❑ audibly perceptible	❑ visually perceptible
In the case of distortion, it is related to:	❑ absent or little tongue-tip trills<br>❑ multiple tongue-tip trills<br>❑ interdental tongue: [ ] anterior [ ] lateral	❑ elevation of the back of tongue<br>❑ lowering of the back of tongue<br>❑ other: ______________________

Trials
Repetition of syllables (request the repetition of syllables containing the altered phones, using the vowel /e/; then, observe if the articulatory production of the altered phone changes when the correct pattern is provided)

**Tested phone**	**The production does not change**	**The production improves**	**The production becomes adequate**
	❑	❑	❑
	❑	❑	❑
	❑	❑	❑

Observation: ____

General aspects [   ] Sum of all scores (best result = 0; worst result = 16)

**Saliva:** (0) swallowed	(1) accumulated in the right and/or left commissure	(1) accumulated on the lower lip	(2) spit	(3) drooled	
**Tongue position in speech:** (0) adequate	(1) on the floor of the mouth	(2) interdental	(2) retracted	(2) low tip and high sides	
**Mouth opening:** (0) adequate	(1) reduced	(1) increased			
**Mandibular movement:** (0) adequate	(1) deviation to R	(1) deviation to L	(1) protrusion		
**Lip movement:** (0) adequate	(1) reduced	(1) exaggerated			
**Tongue movement:** (0) adequate	(1) reduced				
**Articulation:** (0) precise	(1) unsystematic imprecise	(2) systematic imprecise			
**Intonation:** (0) adequate	(1) altered:				
**Rate of speech:** (0) adequate	(1) increased	(1) reduced			
**Pneumophonoarticulatory coordination:**	(0) normal	(1) altered:			
**Resonance:** (0) oronasal balance	(1) hyponasality:	❑ mild	❑ moderate	❑ severe	
(1) laryngopharyngeal	(1) hypernasality:	❑ mild	❑ moderate	❑ severe	

**In the case of change in precision, it is related to:**			
❑ tone	❑ oronasal breathing	❑ malocclusion	❑ quantity of saliva
❑ hearing	❑ muscular fatigue	❑ use of prosthesis	❑ use of medication
❑ rate of speech	❑ anxiety or depression	❑ neurological disorder	❑ decreased mouth opening
❑ others: ____			

University of São Paulo Bauru School of Dentistry
FAPESP São Paulo Research Foundation

Copyright © 2020 by Giédre Berretin-Felix, Katia Flores Genaro, Irene Queiroz Marchesan, and Maria Inês Beltrati Cornacchioni Rehder.
All rights reserved. This clinical assessment tool or any portion thereof may be copied for clinical and academic purposes. Nevertheless, it may not be sold to a third party.

Diadochokinesis [ ] Sum of all scores (best result = 0; worst result = 8) (request 10 seconds of rapid and repeated utterance of each syllable and the trisyllabic sequence)

	Rate		Rhythm	
	**Adequate**	**Inadequate**	**Adequate**	**Inadequate**
**[pa]**	(0)	(1)	(0)	(1)
**[ta]**	(0)	(1)	(0)	(1)
**[ka]**	(0)	(1)	(0)	(1)
**[pataka]**	(0)	(1)	(0)	(1)

Observation: ____________________

Velopharyngeal function [ ] Sum of all scores (best result = 0; worst result = 18)

(0) absent (1) present

**Blow** (the patient must keep lips protruded)**: [ ]**					
**Prolonged utterance** (the patient must sustain the utterance for four seconds)**:**	[ ] [i]	[ ] [u]	[ ] [f]	[ ] [s]	[ ] [ʃ]
**Repetition of phrases:**	[ ] Polly put the pill in the pocket.	[ ] Ted eats tacos.	[ ] Kate kissed the kid.		
	[ ] Billy bought the ball.	[ ] Daisy dates David.	[ ] Gabby gives gifts.		
	[ ] Filip feed the fox.	[ ] Scott has a sister.	[ ] Shyla shares the shampoo.		
	[ ] Victor visits the valley.	[ ] Zack zips up the zipper.	[ ] Jack jokes with Joe.		

Observation: ____________________

Voice [ ] (request the sustained utterance of the vowel /a/)

**Pitch:**	❑ adequate	❑ low	❑ high
**Loudness:**	❑ adequate	❑ loud	❑ quiet
**Type of voice:**	❑ adequate	❑ altered	

Observation: ____________________

Data collected from examinations: ____________________

Requested examinations (reasons): ____________________

Speech-Language Pathology diagnosis: ____________________

Copyright © 2020 by Giédre Berretin-Felix, Katia Flores Genaro, Irene Queiroz Marchesan, and Maria Inês Beltrati Cornacchioni Rehder.
All rights reserved. This clinical assessment tool or any portion thereof may be copied for clinical and academic purposes. Nevertheless, it may not be sold to a third party.

Prognosis: ❑ good ❑ fair ❑ guarded ❑ poor

____
____
____
____
____
____

Therapeutic plan: ____
____
____
____
____
____
____
____
____
____
____
____

Referral to other professionals (reasons): ____
____
____
____
____
____
____
____
____
____

SUMMARY OF THE OROFACIAL MYOFUNCTIONAL CLINICAL EXAMINATION OF THE MBGR PROTOCOL

**EXTRAORAL EXAMINATION** (best result = 0; worst result = 30)	[ ]
Face (best result = 0; worst result = 15)	[ ]
Lips (best result = 0; worst result = 13)	[ ]
Masseter (best result = 0; worst result = 2)	[ ]
**INTRAORAL EXAMINATION** (best result = 0; worst result = 61)	[ ]
Lips (best result = 0; worst result = 5)	[ ]
Cheeks (best result = 0; worst result = 8)	[ ]
Tongue (best result = 0; worst result = 20)	[ ]
Palate (best result = 0; worst result = 8)	[ ]
Palatine tonsils (best result = 0; worst result = 4)	[ ]
Teeth (best result = 0; worst result = 5)	[ ]
Occlusion (best result = 0; worst result = 11)	[ ]
**MOBILITY** (best result = 0; worst result = 68)	[ ]
Lips (best result = 0; worst result = 27)	[ ]
Tongue (best result = 0; worst result = 24)	[ ]
Soft palate (best result = 0; worst result = 4)	[ ]
Mandible (best result = 0; worst result = 13)	[ ]
**SENSITIVITY** (best result = 0; worst result = 65)	[ ]
Tactile (best result = 0; worst result = 55)	[ ]
Pain on palpation (best result = 0; worst result = 10)	[ ]
**TONE** (best result = 0; worst result = 6)	[ ]
Lips (upper + lower) (best result = 0; worst result = 2)	[ ]
Chin (best result = 0; worst result = 1)	[ ]
Tongue (best result = 0; worst result = 1)	[ ]
Cheeks (right + left) (best result = 0; worst result = 2)	[ ]
**OROFACIAL FUNCTIONS** (best result = 0; worst result = 123)	[ ]
Breathing (best result = 0; worst result = 5)	[ ]
Chewing (best result = 0; worst result = 10)	[ ]
Swallowing (best result = 0; worst result = 36)	[ ]
Speech (best result = 0; worst result = 72)	[ ]

Speech-Language Pathologist: ____

University of São Paulo
Bauru School of Dentistry
FAPESP
São Paulo Research Foundation

Copyright © 2020 by Giédre Berretin-Felix, Katia Flores Genaro, Irene Queiroz Marchesan, and Maria Inês Beltrati Cornacchioni Rehder.
All rights reserved. This clinical assessment tool or any portion thereof may be copied for clinical and academic purposes. Nevertheless, it may not be sold to a third party.

CHECKLIST FOR IMAGE DOCUMENTATION

PHOTO DOCUMENTATION

**Body:**	❑ front	❑ back	❑ right profile	❑ left profile
**Mandible:**	❑ base view			
**Face:**	❑ right profile at rest<br>❑ right profile smiling<br>❑ front after the correction of head posture		❑ front of the lower third<br>❑ right profile of the lower third	
**Teeth:**	❑ upper arch	❑ lower arch		
**Occlusion:**	❑ anterior	❑ right side	❑ left side	
**Tongue:**	❑ at rest on the floor of the mouth		❑ protruded	❑ frenulum

VIDEO DOCUMENTATION

**Mobility:**	❑ lips	❑ tongue	❑ mandible	❑ soft palate
**Chewing:**	❑ habitual	❑ questions		
**Habitual swallowing:**	❑ liquids	❑ solids	❑ questions	
**Speech:**	❑ spontaneous	❑ automatic	❑ naming of pictures	❑ reading of words
**Diadochokinesis:**	❑ [pa]	❑ [ta]	❑ [ka]	❑ [pataka]

USP University of São Paulo Bauru School of Dentistry
FAPESP São Paulo Research Foundation
Copyright © 2020 by Giédre Berretin-Felix, Katia Flores Genaro, Irene Queiroz Marchesan, and Maria Inês Beltrati Cornacchioni Rehder.
All rights reserved. This clinical assessment tool or any portion thereof may be copied for clinical and academic purposes. Nevertheless, it may not be sold to a third party.
